# Assessing the impact of transcriptomics data analysis pipelines on downstream functional enrichment results

**DOI:** 10.1093/nar/gkae552

**Published:** 2024-06-29

**Authors:** Victor Paton, Ricardo Omar Ramirez Flores, Attila Gabor, Pau Badia-i-Mompel, Jovan Tanevski, Martin Garrido-Rodriguez, Julio Saez-Rodriguez

**Affiliations:** Heidelberg University, Faculty of Medicine, and Heidelberg University Hospital, Institute for Computational Biomedicine, Heidelberg, Germany; Heidelberg University, Faculty of Medicine, and Heidelberg University Hospital, Institute for Computational Biomedicine, Heidelberg, Germany; Heidelberg University, Faculty of Medicine, and Heidelberg University Hospital, Institute for Computational Biomedicine, Heidelberg, Germany; Heidelberg University, Faculty of Medicine, and Heidelberg University Hospital, Institute for Computational Biomedicine, Heidelberg, Germany; Heidelberg University, Faculty of Medicine, and Heidelberg University Hospital, Institute for Computational Biomedicine, Heidelberg, Germany; Heidelberg University, Faculty of Medicine, and Heidelberg University Hospital, Institute for Computational Biomedicine, Heidelberg, Germany; Genome Biology Unit, European Molecular Biology Laboratory (EMBL), Heidelberg, Germany; Heidelberg University, Faculty of Medicine, and Heidelberg University Hospital, Institute for Computational Biomedicine, Heidelberg, Germany; European Bioinformatics Institute, European Molecular Biology Laboratory (EMBL-EBI), Wellcome Genome Campus, Hinxton, Cambridgeshire, UK

## Abstract

Transcriptomics is widely used to assess the state of biological systems. There are many tools for the different steps, such as normalization, differential expression, and enrichment. While numerous studies have examined the impact of method choices on differential expression results, little attention has been paid to their effects on further downstream functional analysis, which typically provides the basis for interpretation and follow-up experiments. To address this, we introduce FLOP, a comprehensive nextflow-based workflow combining methods to perform end-to-end analyses of transcriptomics data. We illustrate FLOP on datasets ranging from end-stage heart failure patients to cancer cell lines. We discovered effects not noticeable at the gene-level, and observed that not filtering the data had the highest impact on the correlation between pipelines in the gene set space. Moreover, we performed three benchmarks to evaluate the 12 pipelines included in FLOP, and confirmed that filtering is essential in scenarios of expected moderate-to-low biological signal. Overall, our results underscore the impact of carefully evaluating the consequences of the choice of preprocessing methods on downstream enrichment analyses. We envision FLOP as a valuable tool to measure the robustness of functional analyses, ultimately leading to more reliable and conclusive biological findings.

## Graphical Abstract

**Graphical Abstract:**
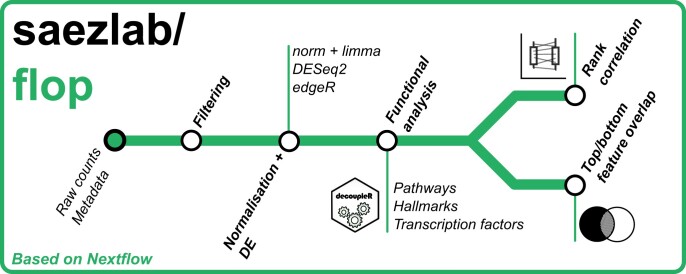

## Introduction

Over the past years, advancements in sequencing technologies have allowed their democratization across the scientific community (1). In particular, RNA-seq offers the possibility to profile entire transcriptomes with nearly complete coverage in a cost-effective manner, and has become the reference technique to analyse gene expression states both at bulk and single-cell levels (2). In the typical workflow to analyse bulk RNA-seq data, sequences are aligned to a reference genome or transcriptome, and the overlapping sequences within regions of interest (such as genes) are counted or estimated (3–5). This results in a count matrix, which is commonly filtered to exclude lowly expressed genes. The counts are then normalized to minimize the influence of technical effects and to make samples comparable. Usually, differential expression (DE) analysis is conducted afterwards, followed by a functional enrichment analysis based on gene sets of processes of interest such as pathways (6,7).

When filtering genes with low expression levels, researchers assume that variance in the measurements for these genes is mostly due to technical noise (8). However, it is also possible that genes in the low expression range still encode valuable biological information, and hence there are different approaches to deal with them. During the filtering step in transcriptomics data analysis, researchers make a choice between coverage and accuracy, yet it remains unknown how this choice impacts downstream analyses.

The normalization step is necessary to eliminate systematic biases that can impact the comparison of gene expression values between samples (9,10). These biases can originate from a variety of sources, such as different library sizes, varying RNA content during different cell cycle phases and the well-studied mean-variance relationship that affects most sequencing technologies. There are multiple methods that have been adapted or developed to carry out the normalization of RNA-Seq count data. Log quantile normalization (11,12) and variance stabilizing normalization (vsn) (13) were initially designed for microarray data but later adapted for RNA-Seq data. On the other hand, methods like the trimmed mean of M-values (TMM) or voom were designed to be used specifically with RNA-seq data (9,10). All DE methods require a previous normalization step, and it has been shown that the choice of a specific normalisation method can affect the resulting DE analysis (14).

The goal of DE analysis is to compare the normalized expression measurements between samples. Also in this step, there are multiple available methods, and three of them are commonly included in most pipelines: Limma (15), edgeR (16) and DESeq2 (17). Limma requires a previous normalization step and models gene expression as a linear function of variables that encode the experimental design. edgeR and DESeq2 directly model count-level data using parametric models that assume an underlying negative binomial distribution of the data. Such models are then used to perform the comparison between samples (9,18).

DE analyses output results for thousands of genes, making direct biological interpretation challenging (19). To reduce dimensionality and enhance interpretability of DE results, genes are usually grouped into functional categories. This step is commonly referred to as functional enrichment analysis or gene set enrichment analysis. These functional categories, also known as gene sets, represent biological processes such as signalling cascades, metabolic pathways or genes that respond to specific chemical or genetic perturbations (20).

Early functional analysis methods primarily focused on over-representation analysis (ORA), which are typically conducted after thresholding DE results to determine which genes significantly change their expression levels. One of the first and most widely used ORA tools was DAVID (21), which provided users both gene set collections and a web application to carry out the analyses. Unlike ORA, Gene Set Enrichment Analysis (GSEA) (22) evaluates the entire gene set instead of individual genes, and does not require a threshold to determine deregulated genes. In recent years, consensus methods have also emerged as a way to combine the results from multiple functional analysis strategies, leading to the development of tools such as Piano (23) or decoupler (24).

The diverse array of alternative approaches for conducting various stages in the downstream analysis of RNA-seq data poses a considerable obstacle in establishing standardized pipelines. To overcome this challenge, benchmark studies (see Supplementary Table S1) were carried out to facilitate the selection of appropriate methods (14,25–33). These benchmark studies employ different strategies to assess method performance, with many utilizing qRT-PCR or simulated data to define a reliable reference for comparison.

Although these benchmarks have provided valuable insights, they focus on comparing individual methods for specific steps and do not investigate the cumulative effects of applying alternative methods in sequence. Secondly, the simulated data used in some benchmarks may inadequately capture the complexities and behaviours observed in real transcriptomic data (34). Lastly, most benchmark studies overlook the influence of method selection on functional analysis outcomes. Consequently, there is a need for consistent standards that help researchers understand how various methods influence data processing and interpretation in transcriptomic analyses (19).

In this work, we developed FLOP (FunctionaL Omics Processing), a nextflow-based (35) workflow that analyses transcriptomic data using multiple combinations of filtering, normalization and DE methods. FLOP then compares the obtained results after downstream functional analysis, providing an easy way of assessing the impact of pipeline choices in the consistency of enrichment results.

To showcase the capabilities of FLOP, we applied it to three collections of datasets studying different biological contexts and with varying sample sizes: Reference of the Heart Failure Transcriptome (ReHeaT), a meta-analysis focused on studying the transcriptomic profiles of patients with end-stage heart failure (36); Cancer Cell Line Encyclopedia (CCLE), a compendium of basal transcriptomic profiles of various types of cancer cell lines across tissues (37), and Pan-cancer Analysis of Chemical Entity Activity (PANACEA), a resource containing the transcriptomic profiles of cancer cell lines perturbed with clinical oncology drugs (38).

Overall, we found that pipeline selection can influence downstream functional analysis results, and that the impact of pipeline choice varies depending on biological context and on comparisons performed. Specifically, we found that filtering of low expressed genes had the greatest impact on the consistency of functional results across pipelines, and for the recovery of expected functional categories. FLOP provides an easy-to-use interface to well-established transcriptomic data analysis pipelines and allows users to explore the effect of pipeline choice on downstream enrichment analyses, typically used as the basis for biological interpretation of results.

## Materials and methods

### Installation, code availability and reproducibility

FLOP can be installed from GitHub (https://github.com/saezlab/flop/) or Zenodo (https://zenodo.org/records/10980523). We packed all the required dependencies in a conda environment which needs to be set up before running the workflow. The exact list of requirements can be viewed in this yaml file (https://github.com/saezlab/flop/blob/main/scripts/flop_env.yaml).

### Data preprocessing

We applied FLOP to eight different datasets (Supplementary Table S2) for which we did not have a clear ground truth: 5 datasets from the ReHeaT resource, the PANACEA DREAM challenge and CCLE. We used 5 datasets from the ReHeaT resource (36): Spurrell19 (58), LiuR (59), Pepin19 (60), Schiano17 (61) and Yang14 (62). These datasets contain information about patients who suffered heart failure, along with other metadata such as age, gender, etc.

We downloaded the PANACEA gene counts from the NCBI GEO portal (GEO accession number: GSE186341) (38). The dataset contains samples from 32 treatments in 11 cell lines. We performed contrasts between each treatment (2 samples) and DMSO (28–60 samples) for each of the 11 cell lines, resulting in 352 comparisons. We translated the gene ID’s from ENTREZ IDs to gene symbols. In multi-mapping situations where several ENTREZ IDs were pointing to the same gene symbol, counts were summed.

The CCLE dataset contains RNA-seq data from 1019 cancer cell lines, which are grouped by 26 parental tissue types (37). We obtained the raw gene counts from the DepMap portal (https://depmap.org/portal/ccle/). We removed tissues with less than 20 cell lines (8 tissues). To account for large differences in cell line number by tissue of origin, we randomly subsetted 20 cell lines per each of the 18 remaining tissues. We then performed all non-redundant pairwise contrasts between all tissue types, resulting in 153 comparisons.

For the benchmarks, we used 7 datasets for the three evaluation strategies presented in this study. Six studies correspond to an unpublished curation effort of public single-cell studies of acute and chronic human heart failure. The count matrix of each study was downloaded from CZ cellxgene, Broad's Single Cell portal or directly from the publication (Supplementary Table S3). For Kuppe2022 and Reichart2022 we converted ENSEMBL gene identifiers to gene symbols using R’s biomaRt v2.58.0. We mapped author's cell-type annotations using regular expressions to a unified ontology that included the following cell-types: Cardiomyocytes (CM), fibroblasts (Fibs), endothelial cells (Endos), pericytes (PCs), vascular smooth muscle cells (vSMCs), myeloid and lymphoid cells. Cells that did not correspond to these annotations were discarded. Pseudobulk expression profiles were generated for each independent sample and cell-type collected in every dataset by summing up the UMI counts of all cells belonging to each of the cell-types defined in our ontology using decoupler-py v1.1.0. We performed contrasts, per dataset, between a given cell type versus a combination of the rest. This way, we computed 42 contrasts (7 cell types × 6 datasets).

The other resource used in the benchmarking strategy corresponds to a single-cell transcriptomics dataset from (50), a collection of perturbational profiles of 15 immune cell types in response to 86 cytokines. We used the package Seurat to compute pseudobulks from the single cell profiles, grouping them by biological context (combination of cytokine + cell type) and biological replicate. We filtered out those pseudobulks that had either less than 10 cells or whose combined sum of read counts was less than 1000. We performed contrasts between PBS and cytokine-treated samples, per cell type. After this filtering, biological contexts involving 4 cell types were discarded, since none of the PBS-treated samples passed the quality control filtering. In total, 944 out of the potential 1290 contrasts (15 cell types × 86 cytokines) passed the quality control filtering.

### Workflow

The count matrix contains genes as rows and samples as columns, and the metadata file specifies the experimental groups to which each sample belongs and the covariates to consider during the filtering and differential expression analysis. Users can also provide a contrast list, which specifies which sample groups to compare. If a contrast list is not provided, all possible non-redundant pairwise comparisons are performed by default.

FLOP integrates several methods for the normalization, differential expression (DE) and functional analysis of RNA-seq data in a modular way. To increase computational efficiency, scalability and reproducibility, we embedded the methods in a Nextflow workflow. The workflow is divided into several processes. The first process downloads the three gene set collections: PROGENy (41), CollecTRI (42) and MSigDB hallmarks (43). The second step carries out the 6 alternative DE analysis pipelines: tmm-limma, vsn-limma, voom-limma, log quantile-limma, edgeR and DESeq2. The next process merges the six pipelines’ output files into a long-format table, which serves as input for the next process, decoupler. After the functional analysis, several consecutive modules merge the results of each dataset. Lastly, the two evaluation modules perform the analysis detailed in following sections: Rank correlation analysis and Top/bottom features overlap analysis.

FLOP output consists of four files: the differential expression results, separated by pipeline and contrast, the activity scores and p-values for all gene sets, and the results for modules 4 and 5. We implemented two profiles to control the parameters for the Nextflow run, depending if the workflow is going to be run on a desktop computer or in a slurm-controlled HPC environment. FLOP also uses a parameter configuration file that can be customized, allowing users to personalize the pipeline runs.

### Filtering strategies

To filter lowly expressed genes, we used the function *filterByExpr*, from the edgeR package, which eliminates genes that are not sufficiently expressed in a minimum number of samples per group. In other words, it takes into account the experimental design and differentiates between genes lowly expressed across groups (probably not biologically relevant) and genes lowly expressed in only some groups but not in others (potentially relevant) [10]. We used the default parameters implemented in this function for all the datasets included in this study (Supplementary Table S4).

To prevent artificial noise in the activity scores of the functional terms, we filtered out contrasts which did not have a sufficient signal, understood as a relevant number of DE genes per contrast, by implementing a cutoff of a minimum of 30 DE genes (this value can be customized).

### Normalization and DE analysis

We selected four methods for the normalization of raw counts (vsn, TMM, log quantile normalization and voom), and three methods for DE analysis (limma, DESeq2 and edgeR). We applied limma on the normalised values, while we applied DESeq2 and edgeR directly on non-normalized counts, since these methods perform an internal normalisation strategy prior to the differential analysis instead. edgeR did not provide *t*-statistics, unlike limma or DESeq2 (we treated Wald statistics as *t*-like statistics). Therefore, we transformed edgeR *F* statistics into t-statistics via the relation *t*^2^ = *F* for a valid comparison between methods. Parameter settings and package versions can be found in the Supplementary Table S4.

### Functional analysis

Decoupler (24) implements multiple strategies to carry out the functional analysis of omics data by combining it with prior knowledge in the form of gene sets. Here, we used *t*-values as input for the analysis. Using the python version of decoupler, we applied the univariate linear model (ulm) method. We used the default threshold of a minimum of 5 genes per gene set.

For the comparisons, we used three prior-knowledge resources: PROGENy (41), CollecTRI (42) and MSigDB hallmarks (43). PROGENy is a prior-knowledge resource that provides a collection of responsive genes for 14 different pathways by analysis of a large set of signalling perturbation experiments. The MSigDB Hallmarks are a collection of 50 independent gene sets that encompass genes which represent a well-defined biological state or process. CollecTRI is a resource that provides relationships between 1186 human transcription factors and their target genes. We downloaded all the gene sets via decoupler (Supplementary Table S4).

For the benchmarks, we additionally used cell type gene sets from PanglaoDB, a resource containing gene biomarkers for 163 cell types. For the hallmark enrichment, we used murine gene sets, since the dataset used was retrieved from mouse models.

### Difference assessment modules

In module 4, we apply Spearman rank correlation to the gene set enrichment results generated by each pair of pipelines and prior-knowledge source.

In module 5, we take the top *N* up-regulated and *N* down-regulated functional categories to calculate the similarity index: for CollecTRI, *N* = 15; for MSigDB hallmarks, *N* = 5; and for PROGENy, *N* = 3. *N* was defined according to each gene set collection size. For genes, the top 5% and bottom 5% of the total number of genes were used as N. The similarity index is calculated using the formula $\frac{{A \cap B}}{N}$, being *A*∩*B* the intersection of two lists of elements, and N the total number of elements in the lists. A similarity score of one indicates that both pipelines have the same top and bottom terms in their ranked lists, whereas a score of zero indicates no overlap between these sections of the ranked lists.

### Evaluation strategies

For the first benchmark, we used a collection of single-cell heart failure studies (44–49) to evaluate whether the methods were able to correctly identify the molecular signatures from a given cell type. In this setting, we defined as true positives those instances in which the enriched cell type gene set, composed by cell-type gene markers, and the cell type as annotated in said datasets were the same. Then, per study, we combined the enrichment results from all contrasts and ranked the instances per activity score. Then, we computed AUROC and AUPRC on the resulting rank using the R package ROCR (63).

For the second benchmark, we used the same studies to evaluate if the different analysis pipelines were able to recover transcription factor (TF) marker activities of each cell-type. We defined as true positives for each cell-type the TFs with the highest binding activity score obtained from chromatin accessibility data as provided in (44). To link TFs to cell types, first, we discarded scores from TFs not present in CollecTRI (for which we would not have enrichment scores). Next, we selected per cell type the 10 TFs with highest binding affinity, and used them as cell type biomarkers (assuming that binding activity from ATAC and TF activity inferred from gene expression are correlated).. Since we did not allow for a TF to be linked to several cell types at once some cell types had <10 markers: 8 for endothelial cells, 5 for pericytes and 3 for vascular smooth muscle cells (vSMCs).

For the last benchmark, we evaluated whether the methods correctly identified molecular signatures in response to cytokines. We used signatures from cytokine perturbations across different immune cell types (50). 44 cytokine treatments were manually linked to 6 different MSigDB hallmarks via the grouping of interleukins in families detailed in (50) (Supplementary Table S5). We performed enrichment analysis on these MSigDB Hallmarks, and then ranked them according to enrichment score. We defined as true positives those instances in which the true cytokine perturbation was linked to the enriched hallmark. We ranked the results per activity scores and computed AUROC and AUPRC.

### Statistical analysis

All *P*-values detailed in the results section were obtained via a one-tailed Wilcoxon rank sum test. We provide FDR corrected p-values in both the text and in FLOP results (64).

## Results

### FLOP: a workflow to compare transcriptomic preprocessing pipelines after functional analysis

**Figure 1.**
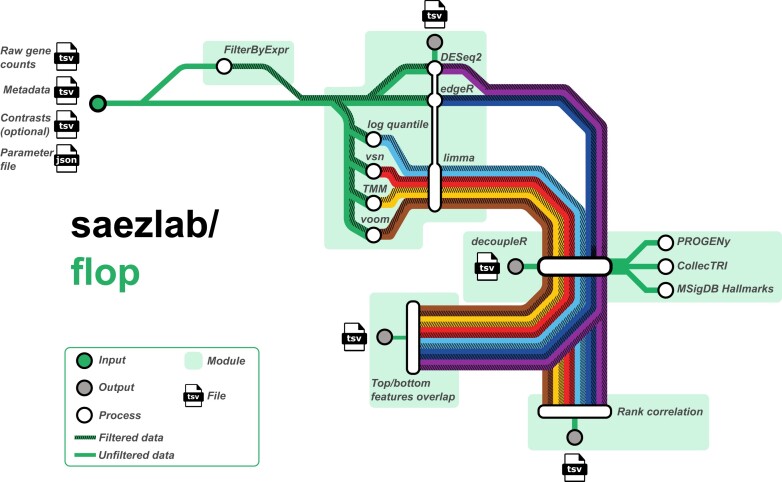
Schematic representation of FLOP methods and modules. From transcript counts and metadata, it uses several differential expression analysis frameworks, performs enrichment analysis and then evaluates the differences in the results via two different metrics.

In this study, we evaluated alternative transcriptomics data analysis pipelines, conceived as combinations of methods that perform the entire processing from raw counts to gene set enrichment scores. Each method input and output are often not directly compatible, making it difficult to combine them for analysis. To address this, we developed FLOP (FunctionaL Omics Processing platform), a unified workflow that takes a count matrix and a metadata file as input and applies alternative processing pipelines to produce functional enrichment scores using different gene grouping categories (Figure 1).

To ensure that the analyses can be reproduced, extended, and scaled effectively, we developed FLOP using Nextflow (35,39). We grouped the methods into three processing modules: (I) filtering, (II) normalization and DE, and (III) functional analysis. We also created two comparison modules (Figure 1). In the first module, the count matrix is either left intact or filtered using edgeR’s function *filterByExpr* with user defined parameters. The second module takes the filtered or non-filtered count matrix as input and applies multiple combinations of normalization and differential expression (DE) methods. We coupled TMM, vsn, log quantile normalization and voom normalization with limma, in addition to edgeR and DESeq2, which employ raw counts as input. This results in a total of 6 combinations of normalization and differential expression analysis tools that coupled to the filtering module creates 12 alternative pipelines.

We unify the output of each individual pipeline in a single table, which contains at least an effect size estimate (e.g. log fold change), a statistical estimate (e.g. *t*-values) and a significance estimate (e.g. adjusted *P*-value) of gene expression changes. As recommended by (40), we use the statistical estimate as input for the functional analysis module. In the third module, we use the univariate linear model (ulm) method from the decoupler package (24), along with three prior-knowledge sources storing different collections of gene sets: signalling pathway footprints (PROGENy, (41)), transcription factor targets (CollecTRI, (42)) and transcriptional hallmarks (MSigDB hallmarks, (43)). We chose the ulm method because its statistical estimates indicate at the same time the direction of the enrichment, positive or negative, and its significance in a single score, and also was observed to be stable and to outperform other methods in a previous benchmark (24).

Following the generation of enrichment scores for each pipeline, contrast, and functional category, we apply the comparison modules. The ‘Rank correlation module’ (Figure 1) assesses the consistency of different pipelines across the complete list of gene sets. To achieve this, the Spearman rank correlation is computed for pairs of pipelines, resulting in a single score that quantifies the similarity in rankings of gene sets within biological contrasts. In the fifth module, denoted as the ‘Top/bottom features overlap,’ we account for the possibility that some researchers may concentrate on the highly up-regulated and down-regulated gene sets for a given contrast. Consequently, this module examines the overlap between the top and bottom sections of the lists through the utilization of a similarity index. The number of features to compare is a user-defined parameter.

### Application of FLOP to transcriptomic studies

**Figure 2.**
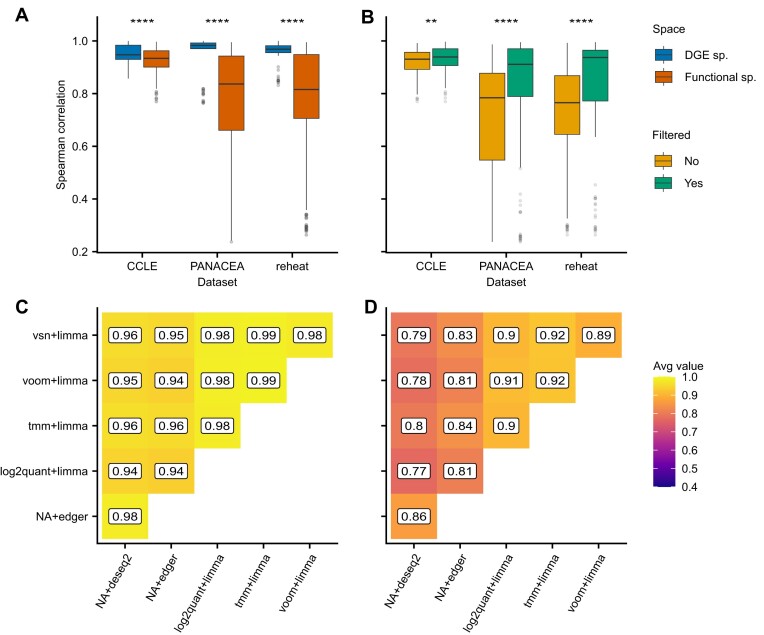
(**A**) Average Spearman rank correlation values across pipeline pairs, per dataset, for both DE and Functional spaces. (**B**) Average Spearman rank correlation values between filtered or unfiltered pipelines versus the rest, per dataset. (**C**) Average Spearman correlation values between filtered pipelines. (**D**) Average similarity scores between filtered pipelines. Statistical analysis was performed via one-tailed Wilcoxon tests for the indicated pairs in panels A and B. *P*-value equivalence: ns: *P* > 0.05, *P ⇐ 0.05, **P ⇐ 0.01, ***P ⇐ 0.001, ****P ⇐ 0.0001. Sd stands for standard deviation in correlation values, sp. stands for space.

To showcase the effect of different pipeline combinations we applied FLOP to three different collections of transcriptomic datasets and compared their results: ReHeaT (36), CCLE (37) and PANACEA (38) (Materials and methods, Supplementary Table S2). We observed that the correlation and similarity values differed greatly between dataset and pipelines (Supplementary Figure S1). Interestingly, correlation values in the differential gene expression (DGE) space were significantly higher than in the functional space across datasets (one-sided Wilcoxon rank sum test, Figure 2A, Supplementary Table S6). We consequently noted a decoupling of the correlation values before and after functional enrichment, suggesting that the choice of an upstream DGE analysis pipeline has an important effect in the functional space despite not being noticeable in the DGE space.

Next, we observed that filtered pipelines showed higher correlation values than the unfiltered ones (one-tailed Wilcoxon rank sum test, Figure 2B), indicating that the removal of potentially noisy genes helps in the convergence of enrichment results. As expected, pairwise comparison of filtered pipelines showed higher levels of agreement than unfiltered ones (Figure 2C, Supplementary Figure S2A). For ReHeaT, unfiltered pipelines coupled to vsn + limma showed the lowest average correlation with other pipelines (average correlation: 0.379). On the other hand, filtered pipelines showed smaller dispersion and higher correlation values (average correlation for filtered pipelines: 0.844, sd 0.188, average correlation for unfiltered pipelines: 0.717, sd 0.206). For CCLE, we found that most Spearman correlation values were well conserved, as supported by the data (min correlation: 0.886 for unfiltered-edger, max correlation: 0.946 for filtered-tmm + limma). However, in PANACEA, we observed a pattern similar to what was previously observed in ReHeaT, where pipelines utilising unfiltered counts displayed lower correlation values (average correlation for filtered pipelines: 0.830, sd 0.208, average correlation for unfiltered pipelines: 0.709, sd 0.205). We attribute this difference to the varying number of samples per group in each dataset and the type of biological signal being analysed, which may result in higher statistical power in CCLE compared to PANACEA.

We also observed a similar trend when looking at the overlap in top-ranked elements between pipelines, although similarity values were in general lower than correlation values, not only between unfiltered pipelines (Supplementary Figure S2B) but also between filtered pipelines (Figure 2D). By examining a specific comparison, we found out that top-ranked terms can show relevant differences even between pipelines which showed overall high correlation (Supplementary Figure S3). While similarity values for CCLE ranged from 0.837 for filtered-tmm + limma to 0.735 for unfiltered-edger, for ReHeaT and PANACEA some pipelines showed much lower values (min similarity value for ReHeaT: 0.459 for unfiltered-vsn + limma, min value for PANACEA: 0.381 for unfiltered-vsn + limma, Supplementary Figure S1)). In other words, focusing on the top up and down regulated gene sets, which is common practice in the interpretation of functional results, revealed a lower level of agreement between pipelines than when the entire lists of gene sets were considered.

Overall, these findings emphasize the potential impact of selecting specific preprocessing methods on downstream functional results, which may not be apparent when only considering the differential expression (DE) space. It further highlights the utility of FLOP in scenarios with limited statistical power, enabling researchers to make more informed decisions regarding pipeline selection.

### Evaluation of methods via cell type-specific transcriptomics profiles

After confirming that the choice of a DGE analysis pipeline can cause differences in the downstream functional space, we aimed to evaluate whether these differences were actually a sign of variable performance. We defined three different benchmark settings with variable expected biological signal, and we evaluated how well each pipeline was able to identify it.

**Figure 3.**
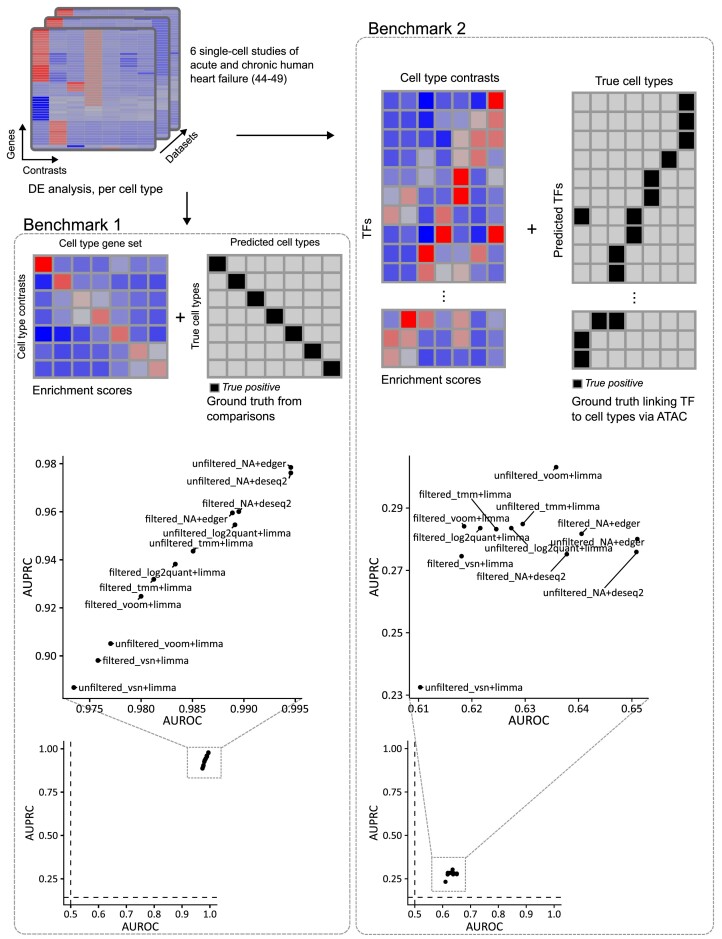
Evaluation strategies using end-stage heart failure transcriptomic studies (44–49). In the first benchmark (left), DGE data from 7 cell types were enriched in cell-type-specific gene sets and compared against the true cell type used to generate the DGE data. In the second benchmark (right), the cell types were linked to TFs via chromatin accessibility data, and then used as ground truth against TF enrichment scores from DGE data. Below, the scatter plots show AUROC/AUPRC values per pipeline, showing the results of the benchmarks based on end-stage heart failure transcriptomic studies. The left plot shows results using cell-type specific gene sets (Benchmark 1), while the right plot shows results using TF enrichment scores linked to cell types (Benchmark 2). Dashed lines show baseline values for AUROC (0.5) and AUPRC (0.143).

First, we used a collection of six single cell datasets (44–49), from which we created cell type-specific pseudobulk profiles. Then, we set contrasts where a specific cell type was compared against a group containing a combination of 6 other cell types, so that the DGE result would correspond to genes solely expressed in said cell type (Materials and methods). Lastly, using cell-type biomarker gene sets, we computed enrichment scores and evaluated the ability of the pipelines to point to the cell type that was being compared (true positive) among the rest (Figure 3A, Benchmark 1). We ranked the cell type gene sets by activity score and computed the Area under the receiver-operating characteristic curve (AUROC) and area under the precision recall curve (AUPRC).

Under these settings, all pipelines showed great performance, with AUROC scores ranging between 0.973 for unfiltered_vsn + limma and 0.995 for both unfiltered_NA + deseq2 and unfiltered_NA + edger (Figure 3, Benchmark 1). Likewise, AUPRC scores ranged from 0.978 for unfiltered_NA + edger to 0.88 for unfiltered_vsn + limma. In general, pipelines containing edgeR and DESeq2 modules showed the best AUPRC values, irrespective of whether they included the filtering module or not (average AUPRC for pipelines including edgeR or DESeq2: 0.969, standard deviation 0.010, average AUPRC for pipelines including limma: 0.923, standard deviation 0.024). Regarding AUROC, these same pipelines, along with unfiltered_log2quant + limma, showed the best performance (average AUROC for pipelines including edgeR or DESeq2: 0.992, standard deviation 0.003, average AUROC for pipelines including limma: 0.981, standard deviation 0.005). While the pipelines using limma clustered at the low end of the score rank, the overall range of values across pipelines, for both AUROC and AUPRC, was very narrow (0.022 for AUROC, 0.091 for AUPRC, respectively).

For the second benchmark, we aimed to evaluate the pipelines in a more challenging scenario. Instead of linking genes to cell types via the cell-type specific gene sets, we used chromatin accessibility data (Assay for Transposase-Accessible Chromatin sequencing, ATAC-Seq) to link TFs to cell types via inferred binding activity (Figure 3, Benchmark 2). Then, instead of performing functional enrichment analysis on the cell type-specific gene sets, we performed TF activity estimation and then evaluated whether the TFs linked to the specific cell type (true positives) showed higher activity than those not. To do this, we ranked the TFs by activity score and computed an AUROC and AUPRC.

Under these settings, all pipelines showed poorer performance than in the previous benchmark (Figure 3). AUROC scores ranged from 0.610 for unfiltered_vsn + limma to 0.651 for unfiltered_NA + edger. Regarding AUPRC, the values ranged from 0.233 for unfiltered_vsn + limma to 0.280 for unfiltered_NA + edger. Pipelines including edgeR or DESeq2 showed higher AUROC values (0.645 for pipelines including them, standard deviation 0.007; 0.623 for pipelines including limma, standard deviation 0.008). However, AUPRC values showed almost no difference between the two groups (0.278 for pipelines including edgeR or DESeq2, standard deviation 0.003; 0.279 for pipelines including limma, standard deviation 0.020). These differences between the metrics may arise from class imbalance, as AUROC tends to be dominated by the majority class. In this benchmark, the true positive to true negative ratio is 1/6.

Under these two settings, pipelines’ differences in performance were not substantial. In the first evaluation setting, all pipelines successfully recovered a signal which was very strong. In the second benchmark, however, using a less reliable prior knowledge resulted in the pipelines showing poor performance, also having small differences between them.

### Evaluation of methods included in FLOP using cytokine-perturbed transcriptomics profiles in immune cells

**Figure 4.**
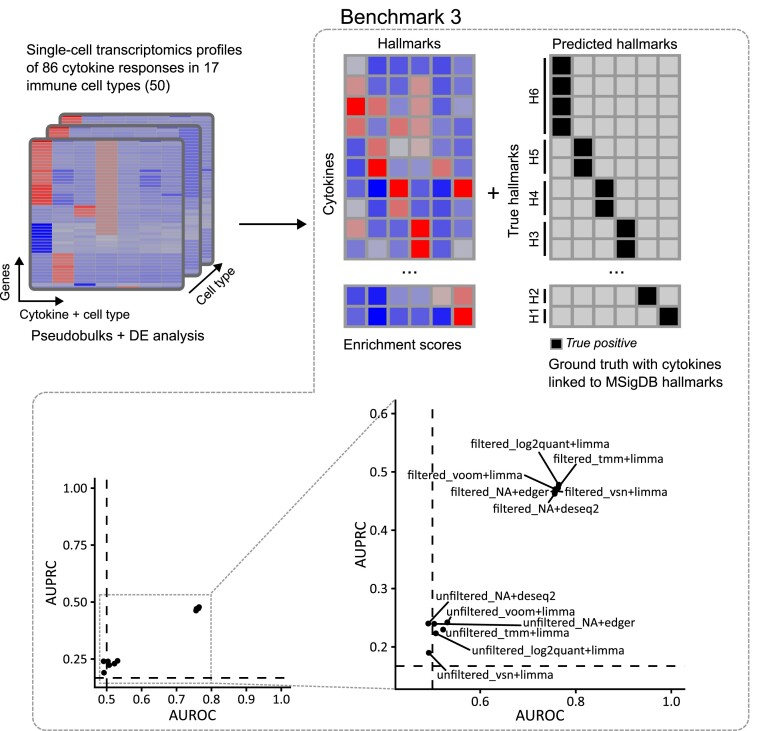
Evaluation strategy using cytokine-perturbed transcriptomic data for different immune cell types (50). DGE data from 86 cytokine treatments and 17 cell types were enriched in MSigDB hallmarks gene sets. The ground truth was generated by linking cytokines to MSigDB hallmarks and compared against the enrichment scores for the different hallmarks. AUROC and AUPRC scores per pipeline were computed. Dashed lines show baseline values for AUROC (0.5) and AUPRC (0.167).

For the last benchmark, we used a single cell transcriptomics dataset (50), where 86 different cytokines were applied to 17 immune cell types (Figure 4). To carry out DGE analysis, we performed contrasts between cytokine-treated samples and DMSO-treated samples per cell type. We then manually matched the different cytokines to suitable MSigDB hallmarks (45 cytokines matched to 6 MSigDB hallmarks, Supplementary Table S4). We computed enrichment scores for the matched hallmarks, and ranked them according to these scores. Like in the previous section, we computed AUROC and AUPRC to assess pipeline performance.

Under these settings, we observed a clear division in performance between pipelines including the filtering module and pipelines lacking it (Figure 4). Filtered pipelines scored higher in both metrics (AUROC: 0.759, sd 0.003; AUPRC: 0.470, sd 0.005) compared to their unfiltered counterparts (AUROC: 0.508, sd 0.160; AUPRC: 0.227, sd 0.020). In addition, in this setting there were no clear differences in performance between pipelines incorporating edgeR or DESeq2 compared to pipelines including limma. These values show that the unfiltered pipelines fail to perform differentially better than a random model, while filtered pipelines show enhanced performance according to both metrics.

In conclusion, the results presented in this study highlight the differences that transcriptomics data analysis pipelines may cause in their outputs. Moreover, complexity reduction using enrichment analysis methods actually exacerbated these differences, instead of reducing them. In one of the three benchmarks, the filtering step was the most important element to reduce these differences and to enhance pipeline performance, while there were no noticeable differences in the other two.

## Discussion

In this study, we present FLOP, a workflow designed to assess the impact of different transcriptomic data analysis pipelines on downstream enrichment analysis results.

We applied FLOP to five studies sourced from ReHeaT, a meta-resource of heart failure transcriptomics data developed by our group, which represents a common clinical application scenario of transcriptomic data analysis. We showcased its capabilities to detect instances of low agreement between pipelines in the gene set space, which might not be present in the differential expression analysis space. We also applied FLOP to cancer cell line data from two distinct studies, namely, CCLE (37) and the PANACEA DREAM challenge (38). These datasets were chosen for their anticipated differences in statistical power due to variations in biological signal and sample size. As expected, the results obtained in the CCLE dataset exhibited higher robustness across different pipeline choices. Conversely, the results derived from the PANACEA dataset displayed much greater variability, both when considering the entire gene set list and when focusing on its extremes.

We also provide three benchmarks to evaluate the performance of the 12 end-to-end pipelines included in FLOP, covering all the processing from raw counts to enrichment scores. The main conclusion derived from our results is that the performance of the methods in the different scenarios was dependent on two main variables: the reliability of the prior knowledge and the intensity of the biological signal, defined as the strength of the biological differences across groups. In the first setting using cell type biomarkers, where both biological signal intensity and the gene set reliability were high, all methods performed equally good. By decreasing the reliability of gene sets by linking TFs to cell types via ATAC-seq, all methods performed equally bad. However, for the third case, where the cytokine perturbations were linked to MSigDB hallmarks, the biological signal was moderate (some cell types were not responsive to the cytokines) but the reliability of the prior knowledge was high. In this case, the benchmark results showed a clear decoupling between filtered and unfiltered pipelines. This provides a first view of how the cumulative effect of normalization, DGE analysis and functional enrichment methods impact the ability of these pipelines to correctly recover biological information.

Numerous benchmarks have investigated the impact of method choice on differential expression (DE) analysis results (14,25–33). However, none of these studies evaluated the impact of method selection on downstream gene set enrichment results. Additionally, various pipelines have been developed to handle both DE analysis and functional analysis tasks. For instance, in (51), authors present a pipeline for pathway enrichment analysis on RNA-seq data. However, it lacks the flexibility to choose from different preprocessing methods. Similarly, the tool described in (52) primarily focuses on network analysis of RNA-seq data but also offers the capability to perform GO overrepresentation analysis. Yet, it provides only limited options in terms of method combinations. In contrast, (53) proposed a pipeline that allows for differential expression analysis using several methods, but it lacks a comprehensive start-to-end workflow to handle the entire process and does not offer a means to effectively compare the methods based on their results.

Putting our findings from benchmarks 1 and 2 in context with previous literature, we found mixed claims. In (26,27), DESeq2 and edgeR were the top performer pipelines among the ones which were included in FLOP. However, in (28), there were no large differences between DESeq2, edgeR and limma-voom, which were outperformed by their own method and other Bayesian approaches. (29) also reports similar findings. In (54), there were no substantial differences when comparing DESeq2, edgeR and limma, and that these differences were largely dependent on the specific properties of the data. However, many of these benchmarks used simulated data, and many of these benchmarks are part of an effort to release a new method, which might bias the benchmark design so that the ‘new’ method outperforms the rest (34).

Regarding the results from benchmark 3, we could not find any previous study that has evaluated the performance of filtered versus unfiltered pipelines. However, (55) report that applying a filtering strategy increased the share of functionally annotated DE genes, which might increase the signal-to-noise ratio and enhance performance, compared to unfiltered pipelines.

This study has some limitations: First, for our applications, we arbitrarily defined certain parameters that can influence the comparison outcomes. Some examples include the minimum number of counts per sample required for the filtering module or the share of top deregulated terms considered when evaluating similarities. In this sense, users should therefore be mindful of the selection of parameter values when running FLOP or any tool in general. Second, due to the combinatorial complexity involved, we focused primarily on preprocessing and employed a single method for enrichment analysis (ulm). However, the assessment of other enrichment methods could be easily implemented through decoupler. While it may come with higher computational costs, it could provide further valuable insights into the interactions of normalization, DE, and functional analysis methods. Third, FLOP includes a handful of popular processing pipelines, among a huge family of tools developed for this purpose (33). Thanks to its modular architecture, new methods could be added to FLOP in the future. Likewise, new evaluation modules could easily be added, in order to accommodate specific needs that researchers might have.

Last, the benchmarking efforts presented in this study lack an absolute ground truth, since the data was obtained from real biological experiments in which not all the confounding factors are known. Although simulated data could be used to evaluate these methods, there has been concern about the generalizability of benchmarking results from simulated to real scenarios (34).

While this study includes pseudobulks from single-cell data, FLOP still cannot handle single-cell transcriptomics data directly. Given the rapidly evolving landscape of single-cell data analysis, it may take some time before it stabilizes enough to support the use of a method like FLOP. While benchmarks evaluating alternative normalization methods for single-cell data are still under active development (39,56), recent efforts have shown that method selection in single-cell analysis can also cause important differences in their results (57).

In summary, we believe that FLOP makes a valuable contribution to the transcriptomics data analysis community. First, we have packed many alternative preprocessing pipelines in a single one-liner workflow, easy to configure and run. Second, we expect FLOP to increase the awareness of the lack of consistency of functional results across the community. FLOP’s metrics are context-independent and easy to interpret, enabling users to quantify the impact of method choices in the results that they report. Third, the evaluation results presented in this study shed light on the interaction between the gene and the enrichment spaces, and empower the community to make informed decisions about the most suitable pipelines for their analyses. Thus, FLOP paves the way for a more robust functional analysis of transcriptomics data.

## Supplementary Material

gkae552_Supplemental_File

## Data Availability

Dataset can be found in Zenodo (https://doi.org/10.5281/zenodo.8272401). It is a small subset of PANACEA containing three treatments and one cell line. The seven datasets used to perform the analysis detailed in this manuscript were also deposited in Zenodo (accession number https://doi.org/10.5281/zenodo.8306225), along with the files containing the prior knowledge sources as to 12.04.2024 (accession number https://doi.org/10.5281/zenodo.10980463). The datasets used for the benchmarking efforts can be downloaded from the corresponding sources, detailed in Supplementary Table S3.
