# Supplementary material for: Assessing the impact of transcriptomics data analysis pipelines on downstream functional enrichment results
Source: Nucleic Acids Res. 2024 Jun 29;52(14):8100–11. doi: 10.1093/nar/gkae552 (PMC11317128; doi:10.1093/nar/gkae552)
Supplement: gkae552_Supplemental_File [file gkae552_supplemental_file.pdf]

Supplementary Materials

Supplementary Figures

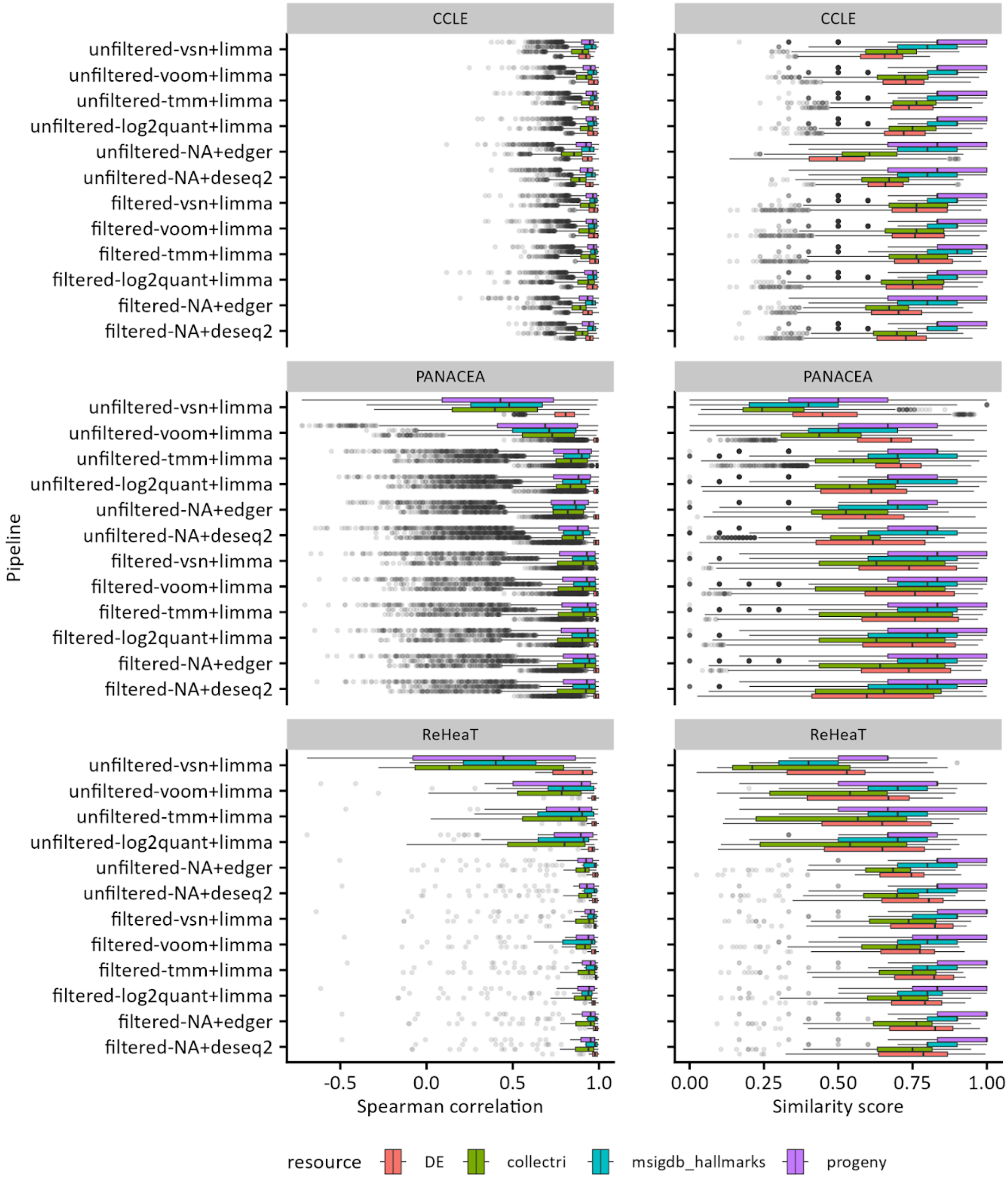

Supplementary Figure 1: Spearman rank correlation and similarity score between pipelines for CCLE, PANACEA and ReHeaT. The plot represents the distribution of correlation values (left) and similarity scores (right) between a given pipeline (y axis) against the rest, for different biological contexts, separated by dataset and prior-knowledge resource.

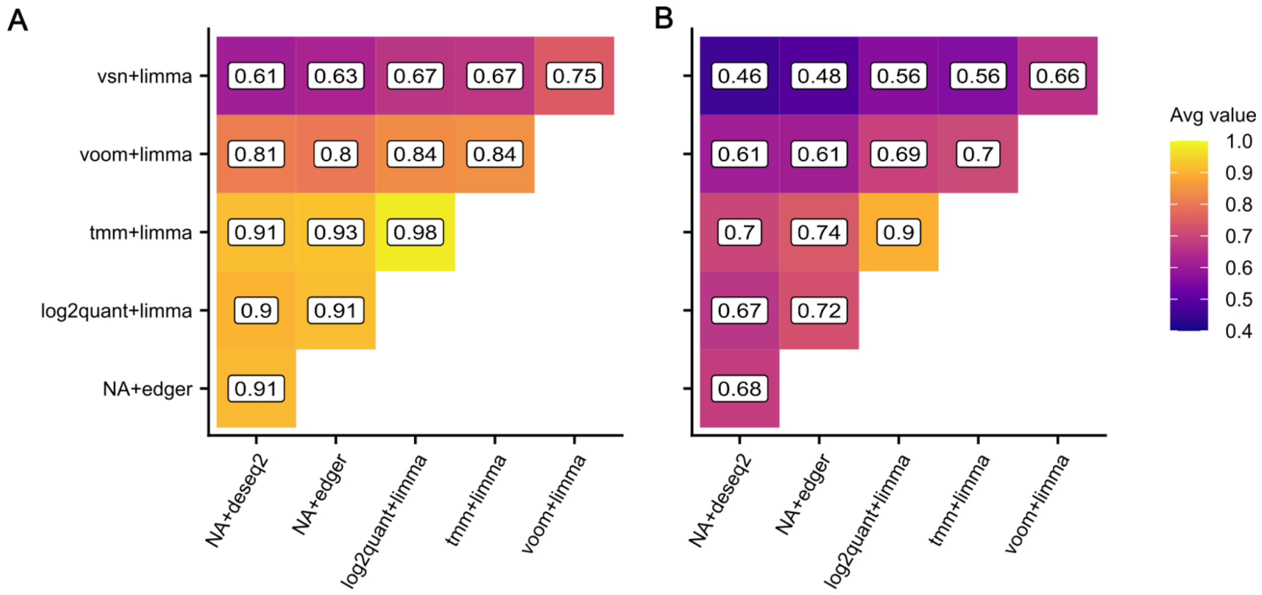

Supplementary Figure 2: Average Spearman correlation values (A) and similarity scores (B) between unfiltered pipelines.

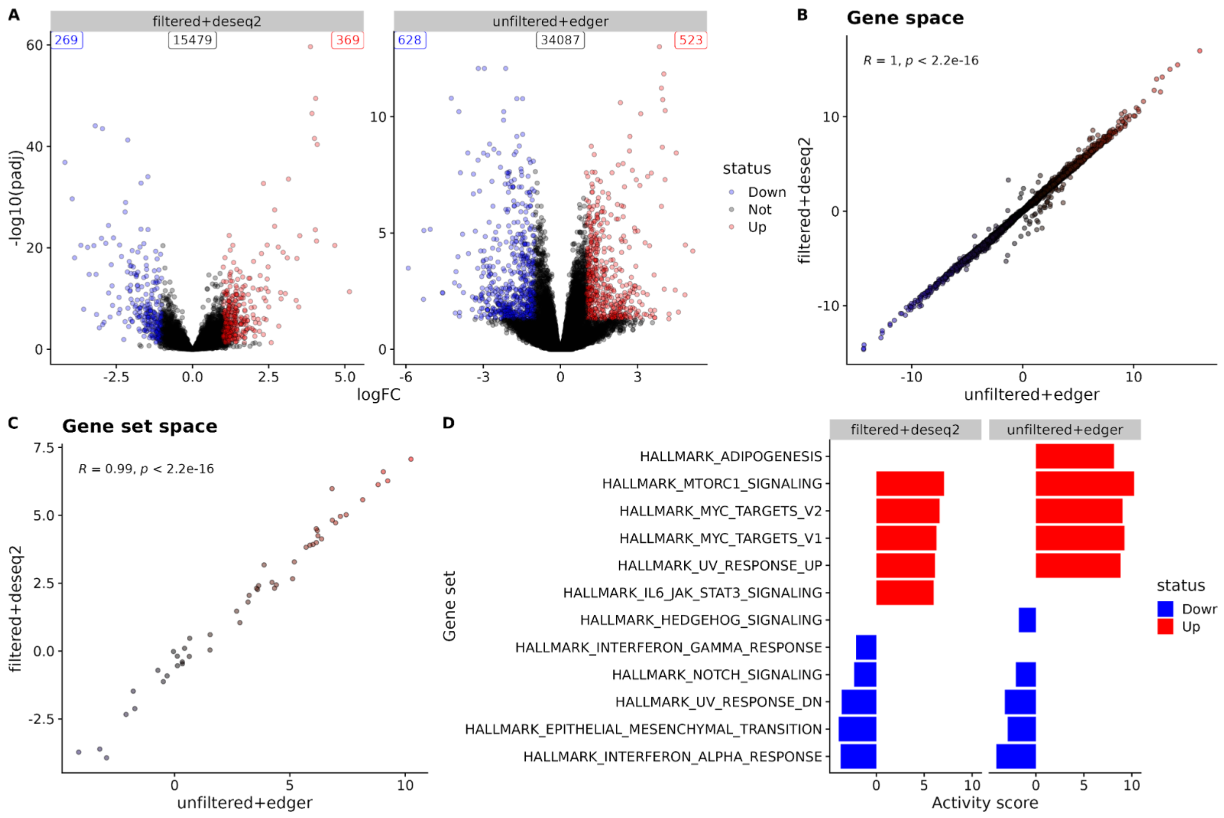

Supplementary Figure 3. Data modalities generated and compared by FLOP. A) Volcano plots depicting the differential expression analysis results for filtered-deseq2 and unfiltered-edger in the Spurrell et al. study (58). All genes with an adjusted P value < 0.05 and an absolute logFC > 1 are highlighted either in red (up-regulated) or blue (down-regulated). The total number of up, down and not regulated genes is displayed in the top corners. B) Scatter plot displaying Spearman correlation between the statistical estimate of both pipelines in the common gene space (each point represents a gene). The Spearman correlation score and its P value are indicated in the top left corner. C) Same scatter plot as in B, but in the gene set space (each point represents a MSigDB hallmark gene set). D) Top 10 up and down regulated gene sets per pipeline. The barplot length indicates the gene set activity score as estimated by the ulm method and the colour indicates the mode of regulation (red and blue for up-and down-regulated terms, respectively).

Supplementary Tables

Supplementary Table 1. ***List of benchmarking studies. The table contains the name, the DOI of the published paper, a short summary of the methodology used and the types of data used in the analysis.***

Evaluated step	Study	DOI	Methods	Datasets	Evaluation strategy
Normalization	(14)	https://doi.org/10.1186/s12859-015-0778-7	Eight non-abundance (RC, UQ, Med, TMM, DESeq, Q, RPKM, and ERPKM) and two abundance estimation normalization methods (RSEM and Sailfish)	Real Illumina high-throughput RNA-Seq of 35- and 76-nucleotide sequences produced in the MAQC project and simulation reads.	Spearman rank correlation of genes between the normalization results from RNA-Seq and MAQC qRT-PCR values for 996 genes.
DE	(25)	https://doi.org/10.1186/s12859-018-2261-8	ALDEX2 family of methods, DESeq2, EdgeR	two simulated RNA-seq datasets and two real datasets (SRP082682, PRJNA277985)	Precision and recall from a contingency table of the simulated state of differential expression (as a binary) compared with the predicted state of differential expression (as a binary). For one real dataset, they computed this per microarray channel and then did the average of both estimates.
DE	(26)	http://dx.doi.org/10.1038/s41467-017-00050-4	Regarding differential expression: DESeq2, limma, edgeR, Cuffdiff, ballgown, sleuth.	15 Illumina and Pacific Biosciences (PacBio) data sets from normal human sample NA1287810, human MCF-7 breast cancer cell, H1 human embryonic stem cell (hESC), and the Sequencing Quality Control Consortium (SEQC) data set.	Compared the detected differentially expressed genes with ground truth from expression changes measured by qRT-PCR: spearman rank correlation and RMSD between log2FC of qRT-PCR vs RNA-seq, AUC-30 scores meaning area under ROC for a false discovery rate of 30 percent.
normalization + DE	(27)	https://doi.org/10.1186/gb-2013-14-9-r95	DESeq, edgeR, limma-QN, limma-voom, PoissonSeq, CuffDiff, baySeq	SEQC study (GSE49712) and ENCODE project	Normalization: they performed hierarchical clustering of samples (if clusters resemble biological differences, the normalization was successful) and then estimated Dunn cluster validity index.<br>DE: ROC analysis using genes previously measured by qRT-PCR as set of true differentiation (cutoff of 0.5 logfc). Also they performed an evaluation of type1 errors via null models (using technical replicate samples). For genes uniquely expressed in one condition, they assessed type II errors by performing an isotonic regression model between signal-to-noise ratio and adjusted p-value.
DE	(28)	https://doi.org/10.1093/nar/gkv806	ROTS, DESeq, DESeq2, edgeR, CuffDiff, limma, BaySeq, NOISeq, PoissonSeq	Spike-in dataset (GSE49712), cancer genome atlas (ccRCC), ccRCC validation dataset (EGAS00001000509)	They used predetermined fold changes to assess the sensitivity and specificity of the methods via ROC/AUC analysis and FDR. They also assessed the usefulness of ROTS to associate certain marker genes with patient outcome via a risk score.
DE	(29)	https://doi.org/10.1371/journal.pone.0232271	edgeR, edgeR.glm, edgeR.rb, edgeR.ql, edgeR.ql.rb, DESeq, DESeq2, voom-tmm, voom.qn, voom.sw, baySeq, bayseq.qn, PoissonSeq, SAMseq, ROTS	SEQC study (GSE49712) as spike-in data, simulated datasets from TGCA kidney renal clear cell carcinoma/normal dataset and inbred mouse dataset (bottomly)	ROC curve, true positive rate and FDR per method, for several simulated datasets containing several combinations of dispersion, percentage of DE genes and presence or absence of weak effect sizes
DE	(30)	https://doi.org/10.1186/1471-2105-14-91	DESeq, edgeR NBPseq, TSPM, baySeq, EBSeq, NOSeq, SAMseq, Shrinkseq, limma-voom, limma-vst	several simulated datasets using a negbinomial distribution, with mean and dispersion estimates from real RNA-seq data. They also tested 2, 5 and 10 samples per condition. Finally, they also used two real datasets (10.1371/journal.pone.0017820)	AUC, false discovery curves for different number of DE genes, bot in one and two directions, and for 5 samples per condition. They also checked type I error control, for pval<0.05, and FDR control, for FDR<0.05. For the real datasets, they compared the number of found DE genes per method and their overlap (venn). No ground truth was available for benchmarking here
DE	(31)	https://doi.org/10.1261/rna.046011.114	DESeq, DESeq2, edgeR, EBSeq, sSeq	Six RNA-seq datasets: Bottomly, Bullard, Huang, Montgomery-pickrell, tuch, Qian, were used to estimate biological paramenters (GLM) to later generate simulated data	TPR, Matthews correlation coefficient and F-measure for several sample sizes and for 100 random samplings,
single-cell normalization + DE	(32)	https://doi.org/10.1186/s13059-020-02136-7	Several methods, starting from a Seurat pipeline, in the following steps: doublet detection, filtering, normalization, feature selection, denoising, dimensionality reduction, and clustering	Two real datasets (GSE79636 via IPSCpower package, data from seqc package) and a simulated one.	Adjusted Rand index, mutual information, silhouette width, precision, recall, AUROC, running time, rate of misclassification (for doublets)
DE	(33)	https://doi.org/10.1371/journal.pone.0190152	Regarding differentia expression: baySeq, DESeq, DESeq2, EBSeq, edgeR, limma-voom, NOIseq, SAMseq	Real Illumina Microarray Quality Control dataset and qrt-PCR as ground truth.	They compared identified DE genes between methods and qRT-PCR ground truth. They counted the true positives and false positives per method. They also computed ROC curve, specificity and FPR from several number of methods

Supplementary Table 2: Overview of the datasets used in this study: number of total samples per contrast, separated by control and treatment (if applicable), number of comparisons and the covariates used in the differential expression and the filtering modules. DCM refers to disease cardiomyopathy, HTx refers to if the biopsy was collected from explanted hearts or after introducing an assisting device.

Dataset		N samples per contrast	Ctrl samples per contrast	Treatment/ Disease samples per contrast	N contrasts	Covariates
ReHeaT	Spurrell19 (58)	33	18	15	1	-
	Liu R (59)	5	3	2	1	Age, gender
	Pepin19 (60)	9	3	6	1	Age, gender, DCM
	Schiano17 (61)	6	4	2	1	-
	Yang14 (62)	24	8	16	1	Age, gender, DCM
PANACEA (38)		30-62	28-60, avg 47	2	352	-
CCLE (37)		40	20 cell lines per tissue		153	-

Supplementary Table 3: datasets used for the benchmark strategies. Cui2024 was used for benchmark strategy 3, while the rest were used for benchmark strategies 1 and 2, as per Figures 3 and 4.

Dataset	Year	Data repository	Data link
Kuppe2022 (44)	2022	cellxgene	https://cellxgene.cziscience.com/collections/8191c283-0816-424b-9b61-c3e1d6258a77
Koenig2022 (45)	2022	GEO	https://www.ncbi.nlm.nih.gov/geo/query/acc.cgi?acc=GSE183852
Chaffin2022 (48)	2022	Single Cell Portal	https://singlecell.broadinstitute.org/single_cell/study/SCP1303/single-nuclei-profiling-of-human-dilated-and-hypertrophic-cardiomyopathy#study-summary
Reichart2022 (46)	2022	cellxgene	https://cellxgene.cziscience.com/collections/e75342a8-0f3b-4ec5-8ee1-245a23e0f7cb/private
Simonson2023 (49)	2023	Single Cell Portal	https://singlecell.broadinstitute.org/single_cell/study/SCP1849/single-nucleus-rna-sequencing-in-ischemic-cardiomyopathy-reveals-common-transcriptional-profile-underlying-end-stage-heart-failure#study-summary
Amrute2023 (47)	2023	GEO	https://www.ncbi.nlm.nih.gov/geo/query/acc.cgi?acc=GSE226314
Cui2024 (50)	2024	Custom platform	https://www.immune-dictionary.org/

*Supplementary Table 4. List of the most relevant functions used in FLOP. Here, we included only the parameters whose values we changed. Parameters which are not specified in the table took default values. We also included the package name and version to which the function belongs. * indicates that weights were only used in those PKs that included them.*

Step	Method	Function	Parameter	Value	Package	Version
Filtering		filterByExpr	-	-	edgeR	3.40.0
		DGEList	-	-	edgeR	3.40.0
Normalisation	TMM	calcNormFactors	method	TMM	edgeR	3.40.0
		DGEList	-	-	edgeR	3.40.0
		cpm	prior.count	3	edgeR	3.40.0
			log	T		
	vsn	justvsn	-	-	vsn	3.66.0
	log quantile	log2	-	-	base	4.2.2
		normalizeQuantiles	-	-	limma	3.54.0
	voom	voom	-	-	limma	3.54.0
DE analysis	limma	lmFit	-	-	limma	3.54.0
		TopTable	number	Inf	limma	3.54.0
			sort.by	none		
	DESeq2	DESeqDataSetFromMatrix	-	-	DESeq2	1.38.0
		DESeq	-	-	DESeq2	1.38.0
		results	-	-	DESeq2	1.38.0
	edgeR	DGEList	-	-	edgeR	3.40.0
		calcNormFactors	-	-	edgeR	3.40.0
		estimateDisp	-	-	edgeR	3.40.0
		glmQLFit	-	-	edgeR	3.40.0
		glmQLFTest	-	-	edgeR	3.40.0
		topTags	n	Inf	edgeR	3.40.0
			sort.by	none		
Functional analysis	decoupler	run_ulm	weight	none*, -	decoupler-py	1.6.0
		get_progeny	organism	human	decoupler-py	1.6.0
		get_collectri	organism	human	decoupler-py	1.6.0
		get_resource	name	MSigDB	decoupler-py	1.6.0
Rank correlation		cor	method	spearman	stats	4.2.2
Top/bottom features overlap		jaccardSets	-	-	bayesbio	1.0.0

Supplementary Table 5: Manual mapping between MSigDB hallmarks and cytokines for the third benchmark setting

MSigDB Hallmark	Cytokines
HALLMARK_IL2_STAT5_SIGNALING	IL2, IL4, IL13, IL7, TSLP, IL9, IL15, IL21
HALLMARK_IL6_JAK_STAT3_SIGNALING	IL6, IL11, IL27, IL30, IL31, LIF, OSM, CT-1, NP, IL12, IL23, ILY
HALLMARK_INTERFERON_ALPHA_RESPONSE	IFNA1, IFNB, IFNE, IFNK, IFNY, IFNL2
HALLMARK_INTERFERON_GAMMA_RESPONSE	
HALLMARK_TNFA_SIGNALING_VIA_NFKB	LTA1/B2, LTA2/B1, TNF, OX40L, CD40L, FasL, CD27L, CD30L, 4-1BBL, TRAIL, RANKL, TWEAK, APRIL, BAFF, LIGHT, TL1A, GITRL
HALLMARK_TGF_BETA_SIGNALING	TGFB1

Supplementary Table 6: adjusted (BH) p-values from a one-tailed Wilcoxon rank sum test for correlation values between DE space and functional space, for each pipeline.

Pipeline	CCLE	PANACEA	ReHeaT
filtered-NA+deseq2	0.049	0.00029	0.0098
filtered-NA+edger	0.049	0.00042	0.0056
filtered-log2quant+limma	0.089	0.00015	7.4e-05
filtered-tmm+limma	0.052	1.7e-05	6.6e-04
filtered-voom+limma	0.049	0.00014	1.1e-05
filtered-vsn+limma	0.076	7.4e-06	0.0011
unfiltered-NA+deseq2	0.0077	1.6e-06	0.0025
unfiltered-NA+edger	0.0066	6.7e-07	0.00052
unfiltered-log2quant+limma	0.062	6.2e-06	9.2e-07
unfiltered-tmm+limma	0.034	1e-06	7e-07
unfiltered-voom+limma	0.0056	1.3e-10	1.3e-10
unfiltered-vsn+limma	0.68	1.3e-10	1.3e-10
